# Evaluation of groundwater pollution in a mining area using analytical solution: a case study of the Yimin open-pit mine in China

**DOI:** 10.1186/s40064-016-2023-x

**Published:** 2016-03-31

**Authors:** Tianxin Li, Li Li, Hongqing Song, Linglong Meng, Shuli Zhang, Gang Huang

**Affiliations:** School of Civil and Environmental Engineering, University of Science and Technology Beijing, Beijing, China; Inner Mongolia Autonomous Region Research Institute of Environmental Science, Huhhot, Inner Mongolia Autonomous Region, China

**Keywords:** Groundwater model, Analytical solution, Flow rate, Open-pit mine, Contaminant migration

## Abstract

**Introduction:**

This study focused on using analytical and numerical models to develop and manage groundwater resources, and predict the effects of management measurements in the groundwater system. Movement of contaminants can be studied based on groundwater flow characteristics. This study can be used for prediction of ion concentration and evaluation of groundwater pollution as the theoretical basis.

**Case description:**

The Yimin open-pit mine is located in the northern part of the Inner Mongolia Autonomous Region of China. High concentrations of iron and manganese are observed in Yimin open-pit mine because of exploitation and pumping that have increased the concentration of the ions in groundwater. In this study, iron was considered as an index of contamination, and the solute model was calibrated using concentration observations from 14 wells in 2014.

**Discussion and evaluation:**

The groundwater flow model and analytical solutions were used in this study to forecast pollution concentration and variation trend after calibration. With continuous pumping, contaminants will migrate, and become enriched, towards the wellhead in the flow direction. The concentration of the contaminants and the range of pollution increase with the flow rate increased.

**Conclusions:**

The suitable flow rate of single well should be <380 m/day at Yimin open-pit for the standard value of pollution concentration.

## Background

With the rapid development of mining, problems related to groundwater in mining areas are becoming increasingly prominent. For example, due to drainage of groundwater, a series of environmental problems, such as pollution of groundwater and decline of aquifer levels, in mining areas have occurred (Dhakate et al. 2008). In order to achieve sustainable development and reach a secure status regarding both quantity and quality of groundwater bodies, some management methods for environmental protection should be introduced, particularly in the prediction of groundwater changes and the control of contamination prior to mining activities (Jiménez-Madrid et al. 2012). It is crucial to accurately understand the factors governing pollutant migration, in order to produce reasonable guidance for environmentally sustainable mining in the future.

In recent decades, groundwater flow and contaminant transport play an important role in the complicate groundwater systems (Regli et al. 2003). According to the Williams (1985) details of site’s geology investigation and hydraulic properties measurement are the main characteristics of groundwater flow. Most previous studies have focused on the trend of groundwater flow and the regularity of contaminant transport. A sharp drop in hydraulic head occurs at the center of the model area, and this generates a cone of groundwater depression and a continuous decline of head, with respect to time, as a result of the high rate of groundwater abstraction (Seyf-Laye et al. 2012). Dawson et al. (2007) introduced a two-dimension numerical model to simulate the movement of polluted water in the sub-grade of a highway and investigate the main factors which influence the movement of contaminants in the highway environment. According to his research, factors that have big influence on the groundwater contamination are the permeability of sub-grade soil, suction-water content characteristics, partitioning coefficient, and sorption capacity of aggregates. Some researchers have focused on the assessment of contamination of groundwater via heavy or toxic metals, nitrate, chromium, and the reactive transport of contaminations from mines to groundwater (Bicalho et al. 2011; Li et al. 2012; Hajhamad and Almasri 2009). A mathematical model was proposed for groundwater pollution in a leaky aquifer system, and this model was applied to describe groundwater pollution in the Taiyuan Basin (Xue et al. 1997). Hoeks (1981) proposed an analytical approach that considered the main properties of the soil system. It is useful of applying this approach to predict contaminants transport in groundwater; however, little quantitative information is available for the soil in terms of homogeneity, dispersion coefficients, and complex interaction processes. Moutsopoulos and Tsihrintzis (2005) researched the turbulent and transient flows in confined porous media and two equations for both short- and long-time conditions were introduced. In addition, the inertia effects and non-Darcy flow conditions also were considered in some analytical solutions (Sen 1989; Moutsopoulos 2007; Wen et al. 2008a, b; Sedghi-Asl et al. 2014; Miracapillo et al. 2014). A mathematical model about contaminant transport due to well injection was developed by Hsieh and Yeh (2014) in a confined aquifer. Potsane et al. (2014) considered macroscopic deterministic models that describe contaminant transport in saturated soils, under uniform radial water flow conditions, through comparisons with numerical solutions.

Xu et al. (2014) established both a groundwater seepage model, and solute transport model, for a factory district and surrounding areas, in Qianan City. The Cr^6+^ have been used to be an index of contamination by researchers which predicted the range of pollution halo and its maximum value. There are some factors influenced the contaminant migration in groundwater such as flow direction, hydraulic conductivity, and the concentration of contaminant at the source (Kjeldsen et al. 1998; Klinck and Stuart 1999). Therefore, it is meaningful to use flow simulation models to research the direction of groundwater flow, distribution of hydraulic heads and flow magnitudes (Banejad et al. 2014). The range of pollutants that exceeds standards can be controlled by a constantly updated groundwater, which was obtained through the flow model and solute transport model that was used in Yishui iron mine in Shandong province (Zhou et al. 2013).

This paper based on the previous research proposed a solute model of pollutant concentration and obtained the analytical solution. The present solute model is able to delineate, analyze, and assess contaminant migration and enrichment during the development of an open-pit mine. The analytical method, in comparison with a numerical solution, is accurate to predict the contaminants migration in the underground water system, and does not require a geological model. It is also more economical than a numerical solution. Therefore, in this study, we used the analytical method to analysis the question, and the model has been validated in its application to the study and prediction of the trend of contaminant flow commences before development in an open-pit mine.

## Study area

**Fig. 1:**
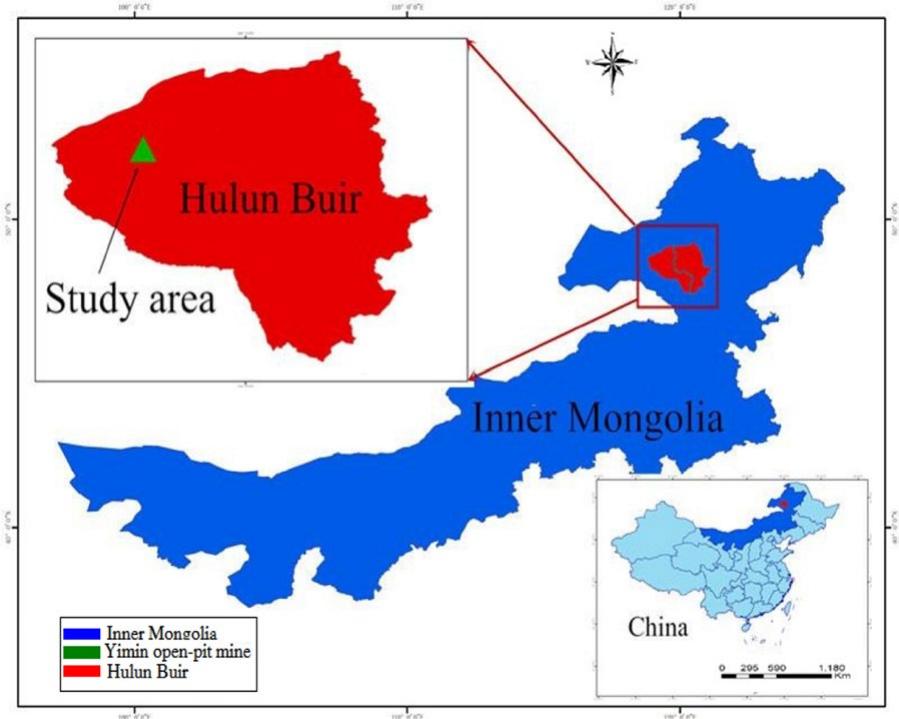
Map of study area

The Yimin open-pit mine is located in the northern part of the Inner Mongolia Autonomous Region of China (Fig. 1). From the existing survey data and groundwater monitoring report, it can be seen that the study area covers 65 km^2^ which is a synclinal basin surrounded by low mountains and hills.

**Fig. 2:**
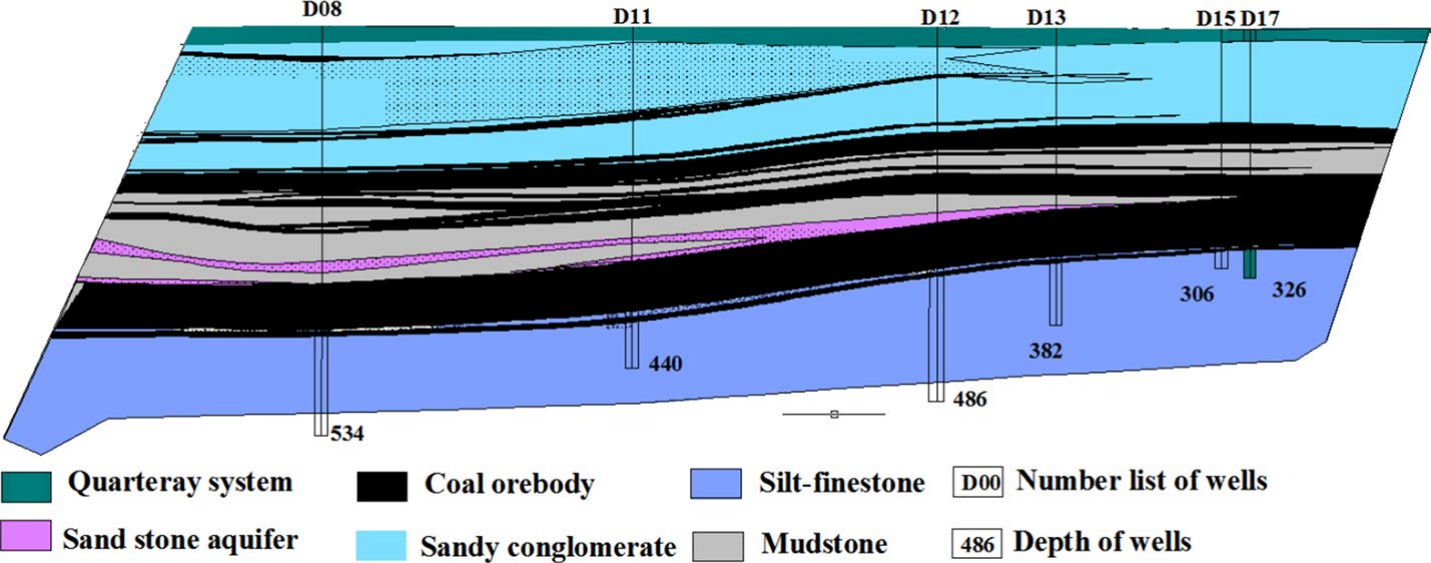
The geological profile and wells location of Yimin coal mine

Quaternary sand and gravel aquifer is a continuous aquifer, it widely distribute above the coal seams and form unconformable contact relationships, the groundwater is in close contact with the coal measure strata water, the lithology of the aquifer consists of sand, gravel, pebbles, coarse sandstone, and medium sandstone, and belongs to the category of porous aquifer. The Tertiary sand and gravel aquifer, which consists of well-sorted sand, gravel, and coarse sandstone, also belongs to the porous aquifer category. The geological profile and some wells’ location was shown in Fig. 2. The coal seams aquifer includes coarse sand and gravel; this aquifer has the strongest hydraulic conductivity and aquosity. In this paper, the water-bearing stratum is generalized in two layers: Phreatic water and Confined aquifer.

According to geological survey reports, the iron minerals in the study area exist in the strata in the form of compounds that are difficult to dissolve. Because of exploitation by open-pit mining in the research area, which caused the groundwater funnel, and the farther the distance from the ground, the lower the oxygen content. This causes a large amount of the dissolved state of the two valence ions of iron to be present in groundwater. This can be presented as follows: FeO + H_2_O + 2CO_2_ = Fe (HCO_3_)_2_.

**Fig. 3:**
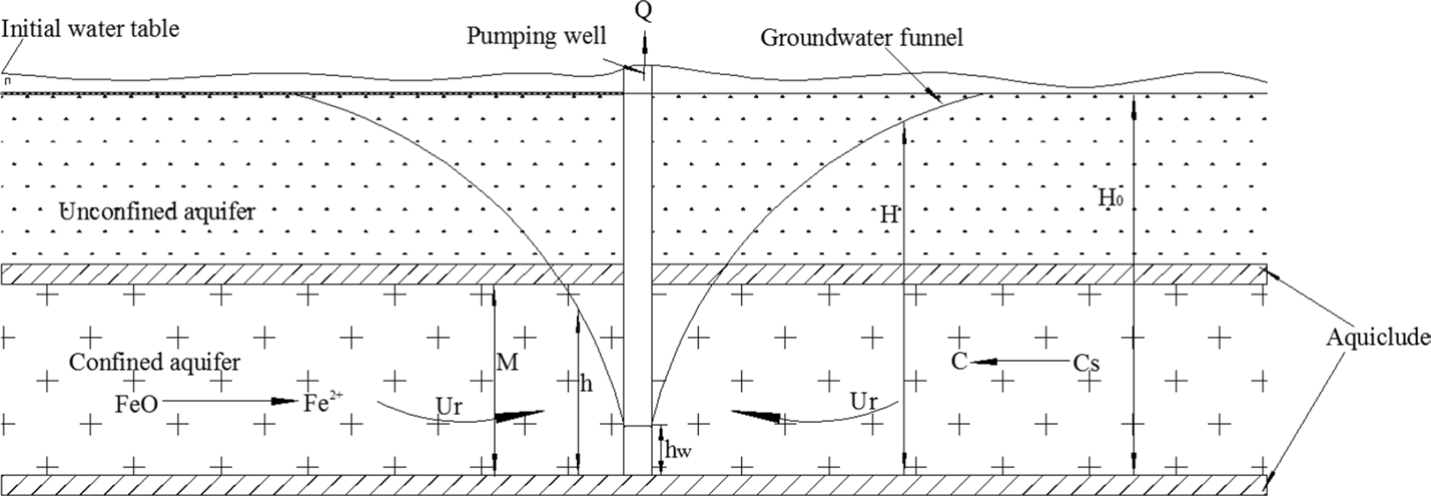
Sketch map of the confined–unconfined groundwater flow and contaminant migration

Considering the geological of bedrock, aquifers in this model are divided into two layers: the upper layer is a phreatic aquifer and the lower layer a confined aquifer with pumping mainly concentrated in this aquifer. The thickness of which is M. A sketch map of groundwater flow and contaminant migration from data obtained from the pumping of a single well is shown in Fig. 3.

## Methods

### Confined–unconfined flow model

With the continuous pumping, the study area of the confined aquifer will be changed into unconfined aquifer which named the confined-unconfined flow. Based on the generalized large well method, a confined–unconfined flow model has been established to simulate the groundwater flow trend in the Yimin open-pit mine and the problem was described as follows with the same assumptions of the previous research (Li et al. 2014):

The governing equation is

1 $$\frac{{T_{m} }}{{\mu_{m} }}\left( {\frac{{\partial^{2} \varphi }}{{\partial r^{2} }} + \frac{1}{r} \cdot \frac{\partial \varphi }{\partial r}} \right) = \frac{\partial \varphi }{\partial t},\quad (r_{0} \le r \le \infty ,t > 0)$$

The initial value and the boundary conditions are as follows:

$$\begin{aligned} \varphi (r,0) & = \varphi_{0} \\ \varphi (\infty ,t) & = \varphi_{0} \\ \mathop {\lim }\limits_{{r \to r_{0} }} 2\pi r\frac{\partial \varphi }{\partial r} & = Q(cons) \\ \end{aligned}$$

where:

$$\varphi_{0} = KMH_{0} - KM^{2} /2;\quad r_{0} = \sqrt {F/\pi } = 0.564\sqrt F$$

*F*: area of mine, m^2^; *H*_0_: initial hydraulic head, m; *K*: permeability coefficient, m/s; *M*: confined aquifer thickness, m; *Q*: flow rate of the whole pumping wells, m^3^/s; *r*: radius of groundwater, m; *r*_0_: reference radius, m; *T*_*m*_: different period corresponding to average hydraulic conductivity, m^2^/s; *t*: time, s; *μ*_*m*_: different period corresponding to average specific yield; $\varphi$: Jilin Gaussian potential function.

### Solute transport model

In this paper, convection, dispersion and chemical reaction will be considered, and the problem will be derived as a stable well flow problem of a planar radial flow field. The following conditions are assumed: (1) the pollutant is mainly distributed in a homogeneous, isotropic and equal-thickness confined aquifer; (2) the aquifer has a complete pumping well, constant flow pumping and an established stable well flow is centered on the well; (3) the diffusion coefficient of pollutants in the medium is assumed to be constant; and (4) pollutants transfer into groundwater from the porous medium by solid phase chemical reactions.

The following contaminant-governing equation can be obtained (Wang 2008):

2 $$\frac{\partial nC}{\partial t} = n \cdot D_{r} \cdot \frac{{\partial^{2} C}}{{\partial r^{2} }} - u_{r} \cdot \frac{\partial nC}{\partial r} - I$$

Therefore, the ordinary differential steady solute-governing equation can be obtained:

3 $$n \cdot D_{r} \cdot \frac{{d^{2} C}}{{dr^{2} }} - n \cdot u_{r} \cdot \frac{dC}{dr} - I = 0$$

where: $I = \lambda \cdot C_{s} \cdot \rho_{b}$. The initial value can be described as,

4 $$C(r)\left| {_{r = L} } \right. = 0$$

Boundary conditions:

5 $$\left. { - n \cdot D_{r} \cdot \frac{dC}{dr}} \right|_{{r = r_{w} }} = f$$

where: *C*: solute concentration in groundwater, kg/m^3^; *Cs*: solute concentration in solid, kg/m^3^; *Dr*: radial dispersion coefficient of porous media, m^2^/s; *r*: radius of groundwater, m; *u*_*r*_: actual fluid velocity, m/s; *r*_*w*_: radius of pumping well, m; *n*: porosity of porous media; *f*: hydrodynamic dispersion, kg/m^2^ s; *λ*: chemical reaction rate constant; *ρ*_*b*_: density of porous media, kg/m^3^.

## Analytical solution

### Analytical solution of flow model

Based on the characteristics of groundwater flow in open-pit mines, an analytical model of special pumping flow transformation from confined groundwater to unconfined groundwater (Li et al. 2014) and velocity solutions can be derived as follows: Boltzmann function transformation (Zhang et al. 2010), equation $u = \frac{{r^{2} }}{4at}$, where $a = \frac{{T_{m} }}{{\mu_{m} }}$. Through substitution, Li et al. (2014) gave the expression of potential function can be presented as,

6 $$\varphi (u) = - \frac{Q}{{4\pi e^{{ - u_{0} }} }} \cdot \int\limits_{u}^{\infty } {\frac{{e^{ - x} }}{x}} dx + \varphi_{0}$$

Hence, the equation of the confined area is

7 $$H = H_{0} - \frac{Q}{{4\pi KMe^{{ - u_{0} }} }}\int\limits_{u}^{\infty } {\frac{{e^{ - x} }}{x}} dx$$

where $$u_{0} = - \frac{{r_{0}^{2} }}{4at}$$, $$\varphi {}_{0} = KMH_{0} - \frac{1}{2}KM^{2}$$.

Based on Darcy’s law, the actual speed in the radial direction can be obtained as follows:

8 $$u(r,t) = - \frac{T}{n}\frac{dH}{dr}$$

where *T*: Confined aquifer conductivity, T = KM.

Substituting Eq. (7) into Eq. (8), the expression of velocity can be presented as

9 $$u_{r} = \frac{Q}{{2\pi ne^{{ - \frac{{r_{0}^{2} }}{4at}}} }} \cdot \frac{{e^{{\left( { - \frac{{r^{2} }}{4at}} \right)}} }}{r}$$

### Analytical solution of Solute transport model

Solving Eq. (3), an equation can be obtained as follows:

10 $$C(r) = \frac{{D_{r} \cdot e^{{\frac{{u_{r} }}{{D_{r} }} \cdot r}} \cdot C_{1} }}{{u_{r} }} - \frac{I \cdot r}{{n \cdot u_{r} }} + C_{2}$$

Based on the boundary conditions, coefficients *C*_1_ and *C*_2_ can be expressed as

$$C_{1} { = }\frac{{D_{r} \cdot I - f \cdot u_{r} }}{{n \cdot D_{r} \cdot u_{r} \cdot e^{{\frac{{u_{r} }}{{D_{r} }} \cdot r_{w} }} }}$$

$$C_{2} = \frac{{f \cdot u_{r} \cdot e^{{\frac{{u_{r} }}{{D_{r} }} \cdot L}} + I \cdot r \cdot u_{r} \cdot e^{{\frac{{u_{r} }}{{D_{r} }} \cdot r_{w} }} - D_{r} \cdot I \cdot e^{{\frac{{u_{r} }}{{D_{r} }} \cdot L}} }}{{n \cdot u_{r}^{2} \cdot e^{{\frac{{u_{r} }}{{D_{r} }} \cdot r_{w} }} }}$$

The expression of concentration can be presented as

11 $$C(r) = \frac{{(f \cdot u_{r} - D_{r} \cdot I) \cdot (e^{{\frac{{u_{r} }}{{D_{r} }} \cdot L}} - e^{{\frac{{u_{r} }}{{D_{r} }} \cdot r}} )}}{{n \cdot u_{r}^{2} \cdot e^{{\frac{{u_{r} }}{{D_{r} }} \cdot r_{w} }} }}$$

Equation (11) expresses the concentration distribution of iron near the pumping well with time and radius.

## Results and discussion

### Solute transport model calibration

According to the exploitation data, the amount of coal mined in the last years is shown in Table 1. From this it can be seen that the production of coal increased from 2007 to 2013. Based on long observed data of hydrology in the study area from the years 2008–2011, the groundwater depression curve derived by the Eq. (1), which is deduced from the model, is more precise, and the calculated value has been validated correctly and may be used to predict the trend of groundwater in future years (Li et al. 2014).

**Table 1:** Exploitation quantity of coal from 2007 to 2014

Year	Exploitation quantity (tons)	Year	Exploitation quantity (tons)
2007	9.41 × 10^6^	2011	1.50 × 10^7^
2008	1.383 × 10^7^	2012	1.53 × 10^7^
2009	1.42 × 10^7^	2013	1.55 × 10^7^
2010	1.453 × 10^7^	2014	1.23 × 10^7^

Based on the iron concentration in 2014 and the data of the geological survey report, some parameter values in this study area were obtained, as listed below in Table 2. The *C*_*s*_ is the initial average value of Iron ion concentration in rock stratum.

**Table 2:** Actual parameters measured in the study area

Symbols	Value	Symbols	Value
*H*_0_ (m)	660	*r*_w_ (m)	0.1
*K* (m/s)	3.72 × 10^−4^	$T_{m}$	0.03
*M* (m)	80	$\mu_{m}$	0.09
*r*_0_ (m)	1009.3	*C*_s_ (mg/l)	0.75
*n*	0.2		

The velocity of transport model was obtained by Eq. (9). Based on the historical records of Yimin open-pit mine, there is no data of chemical ion concentrations before 2014. Therefore, we chose dissolved iron concentration as index to study contaminants transport in this groundwater system. The data of iron ion concentrations near from the artesian wells in 2014 were obtained by experimental tests. The observed data of dissolved iron concentration with the calculated values from analytical solutions are shown in Table 3.

**Table 3:** Comparison of analytical value and real data of dissolved iron concentration in 2014

Samples	Observed data (mg/l)	Calculated values (mg/l)
1	0.81	0.86
2	0.79	0.81
3	0.68	0.69
4	0.79	0.84
5	0.93	0.96
6	3.86	3.90
7	0.38	0.38
8	2.00	2.40
9	2.99	3.10
10	0.63	0.64
11	0.43	0.43
12	0.28	0.30
13	1.17	1.18
14	0.33	0.36

**Fig. 4:**
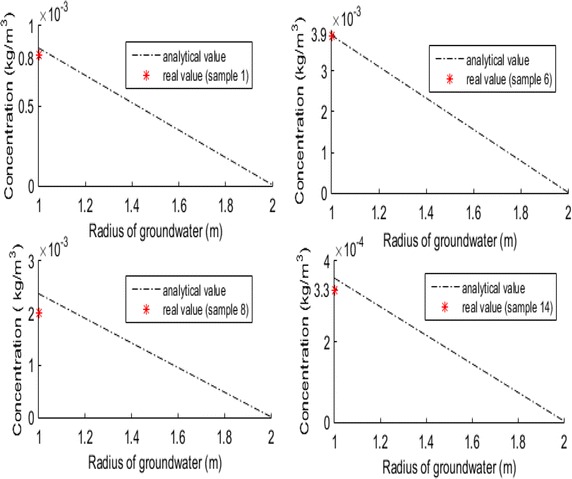
Comparison of analytical value and real data in 2014

From the observed data, the content of iron ions is higher than the standard value of 0.3 mg/l. This study takes samples 1, 6, 8 and 14 as the calibration points. The four samples all located in the mining area, and the sample 1 is from confined aquifer. Sample 6 is from new well and unconfined aquifer. Both sample 8 and 14 are from drinking water well. Basically the four points can reflect the local water quality situation. The single well pumping flow rate is shown in Table 4. The observed data and calculated values from analytical solutions are shown in Fig. 4.

**Table 4:** Single well pumping flow rate

Samples	Flow rate (m^3^/day)	Calculated values (mg/l)
1	432	0.86
6	510	3.90
8	484	2.40
14	389	0.36

From Table 3 and Fig. 4, it can be concluded that the calculated value and real value were very close for the 14 samples of study, and any error within the allowable range. Therefore, the calculated value has been validated and may be used to predict the trend of contaminant ion migration and enrichment in future years with continuous pumping.

### Analysis of the trend of solute concentration with different flow rates

**Fig. 5:**
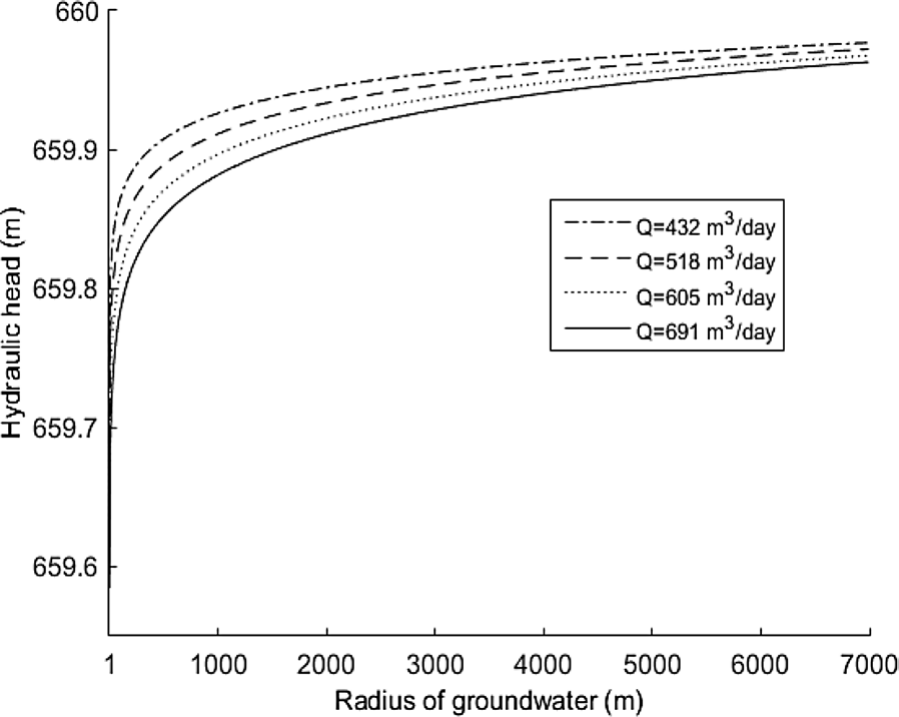
Hydrological head for different flow rates in 2014

This study took the data from 2014 as an example in analyzing the hydrological head for the condition of different flow rate values as shown in Fig. 5. The lower the flow rate, the better the groundwater could be recovered. This illustrates that the pump discharge is relevant to the trend of groundwater change.

**Fig. 6:**
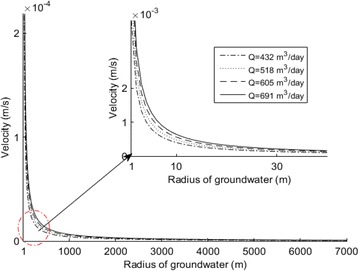
Velocity of different flow rates in 2014

The velocity trend in 2014 with different flow rates can be obtained as shown in Fig. 6. From this figure, the lower the flow rate, the lower the velocity value near the wellhead.

**Fig. 7:**
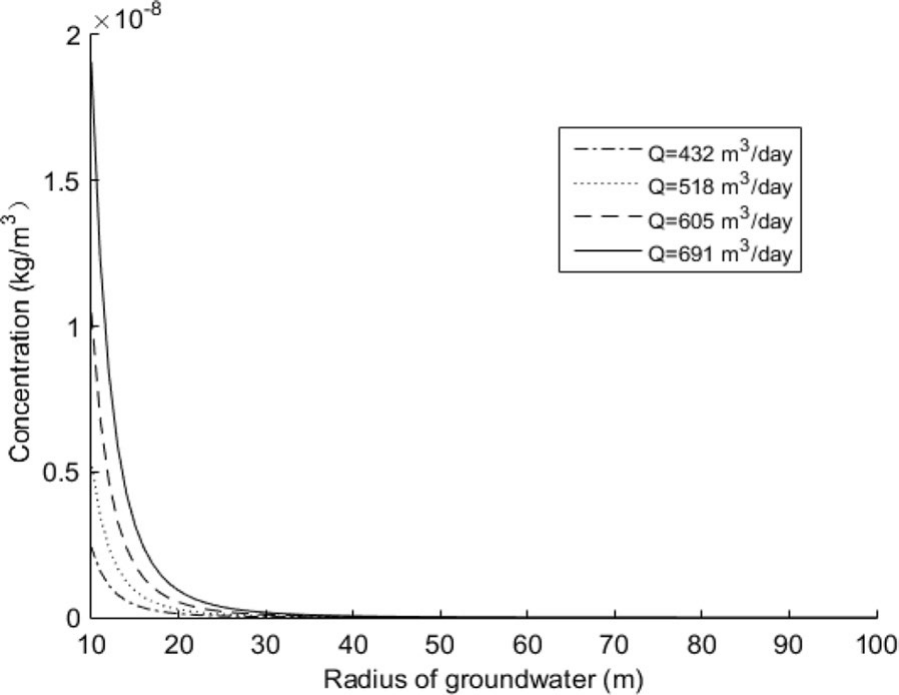
Concentration of different flow rates in 2014

From the Eq. (11), by changing the values of flow rate, the trend of iron ion concentration is shown in Fig. 7. In order to observe the trend easily, the range of radius from 10 m to 100 m is used in this study. In addition, the conclusion can easily be drawn that the lower the flow rate, the lower the value at a radius of 1 m. This can be seen from Tables 5, 6. The value exceeds the standard value of 0.3 mg/l. At *Q* = 432 m^3^/day, the value is 0.8607 mg/l, and at *Q* = 691 m^3^/day, the value is 101.8 mg/l. Therefore, by changing the values of the flow rate it was discovered that the trend of concentration increased while the value of flow rate increased.

**Table 5:** The values of iron concentration when the radius is 1 m with different flow rates in 2014

Flow rate (m^3^/day)	Value (mg/l)
432	0.86
518	4.60
605	22.20
691	101.80

**Table 6:** Sample “1” calculated values in 2014 in the study area

Radius (m)	Values (mg/l)
1	0.86
10	4.56e−6
20	2.39e−7
30	4.45e−8
40	1.37e−8
50	5.47e−9

### Analysis of the trend of solute concentration near the pumping well

**Fig. 8:**
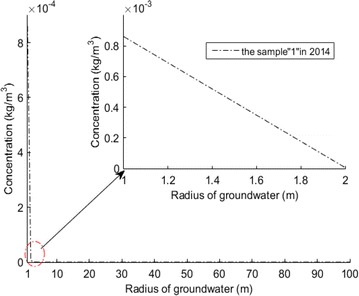
Trend of iron ion migration and enrichment in 2014 near the wellhead

Based on Eq. (11), sample 1 was taken as the study well, so that the trend of solute concentration in the study area in 2014 can be obtained as shown in Fig. 8 and Tables 5 and 6. The figure clearly shows that with continuous pumping, the contaminants will migrate and enrich at the wellhead in the direction of flow. Furthermore, from Fig. 8, it can be concluded that the iron ion concentration is increasing at a higher rate from the radius at the wellhead than the other radius, implying that the rate of increase is increasing with decreasing radius. The acquired solute concentration and groundwater radius in 2014 is shown in Tables 5 and 6. From this figure, it can be seen that at a radius of 1 m, when *Q* = 432 m^3^/day, the value has reached 0.86 mg/l, which exceed the standard value of 0.3 mg/l.

### Prediction of the range of solute concentration with different flow rates

**Fig. 9:**
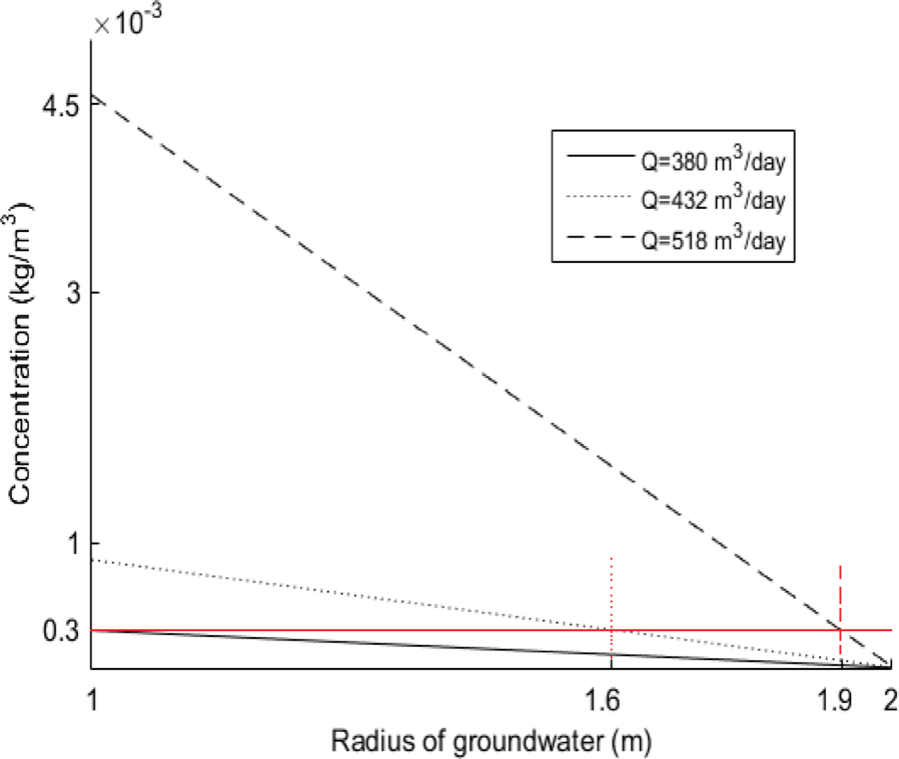
Range of pollution at different flow rates

From Fig. 9, it can also be seen that, with different flow rates, there are different ranges where values exceed the standard value of 0.3 mg/l. When the distance from wellhead is over 1.6 m, the values of iron concentration are higher than standard value of 0.3 mg/l with *Q* = 432 m^3^/day. Also the distance from wellhead is over 1.9 m, the values of iron concentration are higher than standard value with *Q* = 518 m^3^/day. The flow rate not only impacts the value near the wellhead, but also impacts the range of that the value. Most importantly is that when *Q* = 380 m^3^/day, the value at the radius of 1 m is just 0.3 mg/l, that is to say, the flow rate should not exceed 380 m^3^/day when pumping groundwater.

## Conclusion

This paper presents a groundwater flow model and a solute transport model for the confined aquifer of the Yimin open-pit mine. For this purpose, an analytical solution was employed to simulate groundwater flow and solute transport with continuous pumping. The models have been validated correctly and can be used to predict the trend of groundwater in Yimin open-pit mine.

With continuous pumping, contaminants migrate and become enriched towards the wellhead in relation to the flow direction. An increase in flow rate values was found to correspond to an increase in the concentration of contaminants. Finally, we can see that the values of iron concertation don’t exceed the standard value of 0.3 mg/l within the range of 2 m from the wellhead, the flow rate should be 380 m^3^/day. For Yimin open-pit the suitable flow rate should be less than 380 m^3^/day, which may make the iron concentration less than the standard value near the wellhead.

From the discussion above, the model is useful in predicting and studying the trend of groundwater change and contaminant migration before exploitation, and enables further efforts to be made to protect groundwater resources in other open-pit mines with similar hydrogeological conditions.
